# New insight into clinical heterogeneity and inheritance diversity of *FBLN5*-related cutis laxa

**DOI:** 10.1186/s13023-021-01696-6

**Published:** 2021-01-28

**Authors:** Jalal Gharesouran, Hassan Hosseinzadeh, Soudeh Ghafouri-Fard, Yalda Jabbari Moghadam, Javad Ahmadian Heris, Amir Hossein Jafari-Rouhi, Mohammad Taheri, Maryam Rezazadeh

**Affiliations:** 1 Molecular Genetics Division, GMG Center, Tabriz, Iran; 2 Division of Medical Genetics, Tabriz Children’s Hospital, Tabriz University of Medical Sciences, Tabriz, Iran; 3 Department of Medical Genetics, Shahid Beheshti University of Medical Sciences, Tehran, Iran; 4 Department of Otorhinolaryngology, School of Medicine, Sina Medical Research and Training Hospital, Children Medical Research and Training Hospital, Tabriz University of Medical Sciences, Tabriz, Iran; 5 Department of Pediatrics, School of Medicine, Children Medical Research and Training Hospital, Tabriz University of Medical Sciences, Tabriz, Iran; 6 Tuberculosis and Lung Disease Research Center, Tabriz University of Medical Sciences, Tabriz, Iran; 7 Urology and Nephrology Research Center, Shahid Beheshti University of Medical Sciences, Tehran, Iran; 8 Department of Medical Genetics, Tabriz University of Medical Sciences, Tabriz, Iran

**Keywords:** Cutis laxa, FBLN5, Fibulin-5, WES, Autosomal recessive

## Abstract

**Background:**

*FBLN5*-related cutis laxa (CL) is a rare disorder that involves elastic fiber-enriched tissues and is characterized by lax skin and variable systemic involvement such as pulmonary emphysema, arterial involvement, inguinal hernias, hollow viscus diverticula and pyloric stenosis. This type of CL follows mostly autosomal recessive (AR) and less commonly autosomal dominant patterns of inheritance.

**Results:**

In this study, we detected a novel homozygous missense variant in exon 6 of *FBLN5* gene (c.G544C, p.A182P) by using whole exome sequencing in a consanguineous Iranian family with two affected members. Our twin patients showed some of the clinical manifestation of FBLN5-related CL but they did not present pulmonary complications, gastrointestinal and genitourinary abnormalities. The notable thing about this monozygotic twin sisters is that only one of them showed ventricular septal defect, suggesting that this type of CL has intrafamilial variability. Co-segregation analysis showed the patients’ parents and relatives were heterozygous for detected variation suggesting AR form of the CL. In silico prediction tools showed that this mutation is pathogenic and 3D modeling of the normal and mutant protein revealed relative structural alteration of fibulin-5 suggesting that the A182P can contribute to the CL phenotype via the combined effect of lack of protein function and partly misfolding-associated toxicity.

**Conclusion:**

We underlined the probable roles and functions of the involved domain of fibulin-5 and proposed some possible mechanisms involved in AR form of *FBLN5*-related CL. However, further functional studies and subsequent clinical and molecular investigations are needed to confirm our findings.

## Background

Cutis laxa (CL) as a hereditary disorder of skin and connective tissue which can display autosomal dominant (ADCL), autosomal recessive (ARCL), and X-linked recessive (XRCL) inheritance, and also can be acquired [1–3]. The inherited form of CL presents in the early months of life while acquired forms are associated with a late onset presentation, generally in adulthood. This disorder is highly heterogeneous and characterized by loose, redundant, wrinkled and hypoelastic skin as a result of errors in elastic fibers synthesis and structural deficiencies of proteins involved in the extracellular matrix [3, 4]. Since the disease is a connective tissue disorder, its features are associated with multisystem involvement but the precise patho-mechanism of this variable systemic involvement has not been clearly illustrated [5–7]. The prevalence of CL has not been estimated precisely, but the inherited forms of the disease have an incidence of approximately 1 to 2: 400,000 [4].

The ADCL is caused by mutations in structural genes coding for elastin (*ELN*), fibulin-5 (*FBLN5*) and Aldehyde Dehydrogenase 18 Family Member A1 (*ALDH18A1*), and shows ranges of benign clinical variability [8, 9]. Patients are mostly diagnosed in early childhood with loose skin and some systemic involvements (gastrointestinal diverticula, hernias, cardiac and pulmonary complications such as emphysema and bronchiectasis). The manifestations can range from mild to severe but the patients generally have a normal life span in spite of experiencing serious systemic problems like aortic aneurysm [2, 8, 10] (Table 1).

**Table 1:** Diverse clinical features of autosomal dominant and X linked recessive cutis laxa (ADCL and XRCL) associated with each gene ( adapted from OMIM)

Inheritance	ADCL			XRCL
Phenotypes	MIM 123700	MIM 614434	MIM 616603	MIM 304150
Genes	*ELN*	*FBLN5*	*ALDH18A1*	*ATP7A*
SKIN	Loose redundant skin Skin lacks elastic recoil Excessive skin folds No skin hyperelasticity Normal wound healing Skin Histology; Sparse, fragmented elastic fibers	Redundant skin (present at birth, improves over time) Skin folds (present on abdomen and arms) Hyperextensible skin Wrinkled skin (present on backs of hands and wrists)	Thin, translucent skin Lax skin Wrinkled skin	Soft skin Mildly extensible skin Loose, redundant skin Easy bruisability Coarse hair
CARDIOVASCULAR	Mitral valve regurgitation Aortic valve regurgitation	Mitral valve regurgitation	Thin, translucent aortic valve (rare) Aortic insufficiency (rare	Orthostatic hypotension Elongated, tortuous carotid arteries Intracranial arterial narrowing
RESPIRATORY	Emphysema			
HEAD	Premature aged appearance		Microcephaly Triangular face Prominent forehead Broad forehead Prominent ears Low-set ears Congenital cataracts Corneal clouding Strabismus (in some patients)	-Persistent, open anterior fontanel Long, thin face High forehead Long philtrum Hooked nose High-arched palate Long neck
GENITOURINARY	External Genitalia (Male); Inguinal hernia		Unilateral renal agenesis (rare)	Hydronephrosis Ureteral obstruction Bladder diverticula Bladder rupture Bladder carcinoma
MISCELLANEOUS	Genetic heterogeneity Onset of skin manifestations from birth to puberty	One African American female has been described Cutaneous manifestations significantly improved over the first decade of life		
SKELETAL		Scoliosis	Osteopenia (in some patients) Delayed closure of fontanels Wormian bones (in some patients) Abnormal spine curvature (in some patients) Hip dislocation Joint hyperlaxity Adducted thumbs Clenched fingers Clubfoot (in some patients) Pes calcaneovalgus (rare)	Joint laxity Osteoporosis Occipital horn exostoses Kyphosis Mild platyspondyly Coxa valga Pelvic exostoses Short humeri Genu valgum Limited elbow extension Limited knee extension Capitate-hamate fusion Pes planus
ABDOMEN		Extensive folding and redundant skin (present at birth)	Hernias Feeding difficulties	Chronic diarrhea Hiatal hernia
NEUROLOGIC			Psychomotor retardation Hypotonia Brisk reflexes Cranial vessel tortuosity Foramen magnum stenosis (in some patients) Autism spectrum disorder (rare)	Low-normal IQ
Other			Prenatal and postnatal growth retardation	Narrow shoulders Narrow chest Short, broad clavicles Pectus excavatum Pectus carinatum Short, broad ribs

The ARCL is the most common and variable form of CL which is subsequently divided into nine subtypes based on genetic and clinical characterizations. There are various ARCL-associated genes such as the *FBLN5*, EGF containing fibulin extracellular matrix protein 2 (*EFEMP2* also known as *FBLN4*), latent transforming growth factor beta binding protein 4 (*LTBP4*), ATPase H + transporting V0 subunit a2 (*ATP6V0A2*), pyrroline-5-carboxylate reductase 1 (*PYCR1*), ATPase H + transporting V1 subunit E1 (*ATP6V1E1*), ATPase H + transporting V1 subunit A (*ATP6V1A*), aldehyde dehydrogenase 18 family member A1 (*ALDH18A1*) and PYCR1. This type of CL is often a life threatening, generalized neonatal disorder with severe systemic manifestation such as severe gastrointestinal, cardiopulmonary, and urinary abnormalities alongside with the skin manifestations which are presented in the whole body [2, 4, 11] (Table 2).

**Table 2:** Diverse clinical features of autosomal recessive cutis laxa (ARCL) associated with each gene ( adapted from OMIM)

INHERITANCE	ARCL								
Phenotypes	IA; MIM 219100	IB; MIM 614437	IC; MIM 613177	IIA MIM 219200	IIB MIM 612940	IIC MIM 617402	IID MIM 617403	IIIA MIM 219150	IIIB MIM 614438
Genes	*FBLN5*	*FBLN4*	*LTBP4*	*ATP6V0A2*	*PYCR1*	*ATP6V1E1*	*ATP6V1A*	*ALDH18A1*	*PYCR1*
Skin	Loose redundant skin Excessive skin folds Normal wound healing No skin hyperelasticity Increased vascularization, reduced collagen bundle size Underdeveloped elastic fibers in dermis	Velvety skin Normal scarring Collagen bundles smaller than normal Vascularization increased in upper dermis Underdeveloped elastic fibers, severe	Skin laxity	Loose redundant skin Excessive skin folds Abnormal, broken, shortened elastic fibers Decreased amount of elastin Sparse, brittle hair Coarse hair	Cutis laxa Loose redundant skin (especially of dorsum of hands and feet and anterior abdominal wall) Reduced skin elasticity Wrinkly skin Prominent veins	Generalized skin wrinkling Reduced elastic fibers Fragmented elastic fibers Loosely packed collagen fibers Variable diameters of collagen fibers	Generalized skin wrinkling Reduced elastic fibers Fragmented elastic fibers Loosely packed collagen fibers Variable diameters of collagen fibers	Thin, translucent skin Prominent superficial blood vessels due to thin skin Reduced number of elastic fibers Thin or fragmented elastic fibers Degenerated elastic fibers Sparse hair	Skin laxity
Cardiovascular	Supravalvular aortic stenosis Vascular tortuosity Ascending aortic aneurysm	Thickened myocardium (rare) Bradycardia (rare) Aortic aneurysm Pulmonary artery aneurysm Arterial aneurysms, multiple Arterial tortuosity, general Venous tortuosity Arterial stenoses, multiple Vascular fragility Vascularization increased in upper dermis	Pulmonary artery stenosis Patent foramen ovale		No vascular tortuosity	Severe dilation of ascending aortic root Moderate biventricular hypertrophy Mild dilation of right ventricle Reduced diastolic compliance of right ventricle Hypoplastic right ventricle Tricuspid valve stenosis Tricuspid insufficiency Aortic insufficiency Mitral valve prolapse Hypoplastic pulmonary artery Atrial septal defect Incomplete right bundle branch block	Atrial septal defect Dilated ascending aorta Tortuous aortic arch Cardiac failure Cardiomyopathy, hypertrophic Hypertrophy of interventricular septum, mild Long QT interval on electrocardiography (ECG) Incomplete right bundle branch block (ECG)		
Respiratory	Recurrent respiratory infections Emphysema	Emphysema	Laryngomalacia Tracheomalacia Bronchomalacia Emphysema Hypoplastic lung			Laryngomalacia Bilateral pneumothorax (in early infancy)			
Head	Microcephaly Sagging cheeks	Microcephaly (rare) Prominent forehead Prominent premaxilla Micrognathia Dysplastic ears Low-set ears Prominent eyes Small palpebral fissures Downslanting palpebral fissures Hypertelorism, mild Bulbous nasal tip (in some patients) Hooked nose (in some patients) Depressed nasal bridge (in some patients) High-arched palate	Wide fontanels Micrognathia Flat midface Receding forehead Periorbital swelling Hypertelorism Wide nasal bridge Long philtrum Retrognathia	Microcephaly Midface hypoplasia Long philtrum Flat face Low-set ears Downslanting palpebral fissures Strabismus Myopia Short nose Anteverted nares Small mouth High-arched palate Dental caries	Microcephaly Large fontanel Broad, prominent forehead Sagging cheeks Aged appearance Triangular face Midface hypoplasia Prominent ears Blue sclerae Downslanting palpebral fissures (in 2 patients) Hypotelorism (in 2 patients) Deep-set eyes (in 2 patients) Prominent bulbous nose	Progeroid facies Mask-like triangular face Short forehead Long philtrum Prominent nasolabial folds Short pointed chin Low-set ears Misfolded helices Hypertelorism Entropion Nystagmus Prominent beaked nose High nasal root Broad nasal tip Broad columella Narrow nostrils High-arched palate Dental crowding	Progeroid facies Mask-like facies Triangular face Short forehead Receding chin Low-set ears Large ears Prominent ears Simple folded helices Hypertelorism Entropion Blepharophimosis Downslanting palpebral fissures Bilateral cataract Bulbous nose Broad nasal bridge	Brachycephaly Prominent forehead Large fontanelles Progeroid appearance Low-set ears Large ears Corneal opacities Cataracts Hypotelorism Hypertelorism Strabismus Myopia Salt-and-pepper retinopathy (in some patients) Pinched nose Hypoplastic alae Small mouth	Corneal opacification
Genitourinary	External Genitalia (Male) Inguinal hernia External Genitalia (Female) Inguinal hernia Bladder Bladder diverticula	External Genitalia (Male) Inguinal hernia	External Genitalia (Male) Inguinal hernia Kidneys Hydronephrosis Bladder Bladder diverticula			External Genitalia (Male) Inguinal hernias, bilateral Internal Genitalia (Male) Cryptorchidism, bilateral	External Genitalia (Male) Micropenis Inguinal hernia Internal Genitalia (Male) Cryptorchidism, bilateral	Internal Genitalia (Male) Undescended testes (in some patients)	
Miscellaneous		Relatively mild cutis laxa, associated with severe vascular abnormalities Massive aortic aneurysm can cause airway compression in affected infants		Skin abnormalities tend to decrease with age		One Kuwaiti and one Iranian family with 2 sibs each have been reported (last curated March 2017) Variable congenital heart defects	Based on 3 patients (last curated March 2017) Variable cardiac and skeletal features may be present		
Skeletal	Congenital fractures Joint laxity Arachnodactyly	Joint hypermobility, generalized Fractures at birth Arachnodactyly Contractures of third to fifth fingers Arachnodactyly	Joint laxity Wide sutures Widely spaced first and second toes Plantar crease	Joint hyperextensibility Large anterior fontanel Delayed closure of the fontanel Congenital hip dislocation	Joint hyperextensibility Osteopenia Scoliosis (in 2 patients) Congenital hip dislocation Bowing of long bones Long digits (in 2 patients) Clasped thumb	Joint laxity Recurrent dislocations of temporomandibular joint Kyphoscoliosis Hip dysplasia Flexion contractures of knees Clenched hands Ulnar deviation of fingers Flat feet Club feet	Flexion contractures of all joints Dislocated hips Camptodactyly Club feet	Delayed bone age Hyperextensible joints Dislocated joints Wormian bones Wide cranial sutures Scoliosis Congenital hip dislocation Adducted thumbs Clenched fists Talipes equinovarus Pes calcaneovalgus	Congenital hip dislocation Patent anterior fontanelle Joint laxity -Scoliosis
Abdomen	Umbilical hernias		Umbilical hernia Gastroesophageal reflux Diverticula Pyloric stenosis Intestinal dilatation, tortuosity Rectal prolapsed	Feeding problems in infancy	Gastroesophageal reflux		Inguinal hernia Umbilical hernia		Hernias
Neurologic		Hypotonia Brain hemorrhage		Delayed motor development Mental retardation Seizures Hypotonia Partial pachygyria Cobblestone lissencephaly, posterior frontal and parietal regions Board and poorly defined gyri Polymicrogyria Dandy-Walker malformation	Developmental delay Agenesis of the corpus callosum (in 2 patients) Hydrocephalus (in 2 patients)	Hypotonia	Hypotonia Seizures Speech delay Motor delay Enlarged ventricles with white matter involvement Periventricular parietooccipital gliosis Diffuse thickening of cerebral cortex Thin corpus callosum	Developmental delay Hypotonia Athetoid movements Hyperreflexia Seizures Grimacing	-Delayed motor development -Mental retardation -Hypotonia -Athetoid movements
Other	Fetal overgrowth Pectus excavatum Diaphragmatic hernia	Fetal overgrowth (in some patients) Pectus excavatum Hypoplastic diaphragm Diaphragmatic hernia	Postnatal growth delay Diaphragm hernia or eventration Low muscle tone	Intrauterine growth retardation (IUGR) Failure to thrive Hypotonia Lipodystrophy Abnormal distribution of subcutaneous fat	Intrauterine growth retardation Failure to thrive Poor postnatal growth	Short stature Low weight Marfanoid habitus Sparse subcutaneous fat Marked muscular atrophy Reduced muscular strength	Failure to thrive Marfanoid habitus Abnormal fat distribution	Intrauterine growth retardation (IUGR) Failure to thrive Poor postnatal growth Pectus excavatum	Intrauterine growth retardation Postnatal growth delay

Patients with XRCL also known as Occipital horn syndrome show distinct phenotypic features at birth such as hyperextensible wrinkled skin with droopy faces, occipital horns, neurologic defects, urinary tract infections, bladder diverticulae, orthostatic hypotension, inguinal hernias and diarrhea [12, 13]. This form of CL is caused by mutation in *ATPase copper transporting alpha* (*ATP7A*) gene which is involved in copper secretion from nonhepatic tissues, copper absorption from the small intestine, and copper transport across the blood–brain barrier [5, 13, 14] (Table 1).

Here, we present a family with ARCL type-IA in their members as a result of a novel mutation in the *FBLN5* gene. We also propose some explanations for phenotype heterogeneity and suggest some possible mechanisms of CL pathogenesis resulting from different mutations in the *FBLN5* gene.

## Material and methods

### Patients

**Fig. 1:**
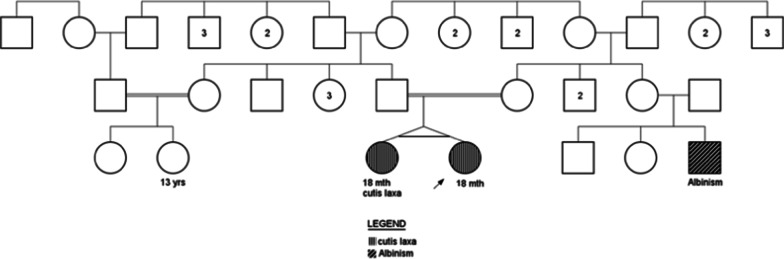
Family pedigree of the patients and their relatives representing autosomal recessive CL in a large family

**Fig. 2:**
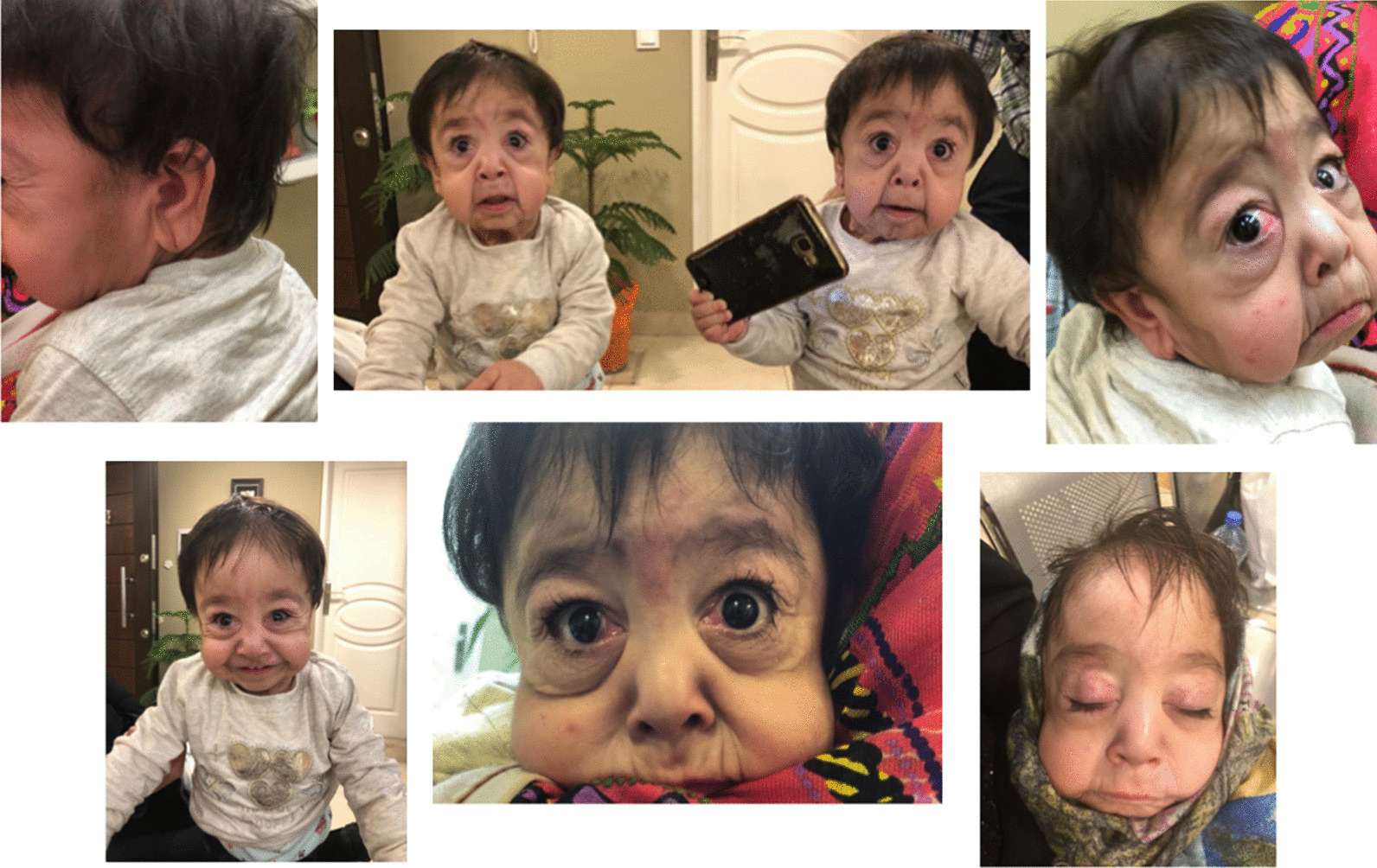
Phenotypic characteristics of one of the twins. Abnormal facial features such as premature aging appearance, wrinkled skin, sagging cheeks and big eyes are the most striking findings that have been presented in the pictures

Two-year-old monozygotic twin sisters whose parents were consanguineous and had experienced about a 10-year infertility, were referred to GMG center for genetic analysis (Fig. 1). These twins have been conceived through in vitro fertilization (IVF). When they were 7 months, old wrinkled, loose and sagging skin appeared on their whole body specifically on groins, necks, armpits and faces. Additionally, physical examination indicated excessive growth of facial and body hair, sparse eyebrows, big eyes, dysplastic ears and premature aging appearance (Fig. 2). Extra investigations for systemic involvements did not reveal any pulmonary complications, gastrointestinal and genitourinary abnormalities, but one of them was diagnosed with ventricular septal defect (VSD). The study protocol was approved by Ethical Committee of Shahid Beheshti University of Medical Sciences and all methods were performed in accordance with the relevant guidelines and regulations. Informed written consent forms were obtained from study participants.

The patients’ parents and relatives were phenotypically normal and did not have symptoms of connective tissue disorders or multiple congenital anomalies in their children with the exception of the probands' cousin who had albinism.

### Molecular genetic studies

We got written informed consent from the parents and their relatives for genetic analysis and publication of the patients’ photos. This study was approved by the ethical committee of Shahid Beheshti University of Medical Sciences. Genomic DNA of patients and their family members was isolated from peripheral blood lymphocytes using DNA extraction kit (GeneAll Exgene Blood SV Mini). Initially, whole exome sequencing (WES) was performed in one of twin sisters to identify genetic bases of CL in this family. Once the variant has been detected, specific primers including 5′-AGAAGAATCCTGGGCAGTGG-3′ as forward primer and 5′-CGCATAGCAAGGTTCAGGTC-3′ as reverse primer were designed for subsequent co-segregation analysis of the other sister and family members.

## Results

### Molecular genetics results

**Fig. 3:**
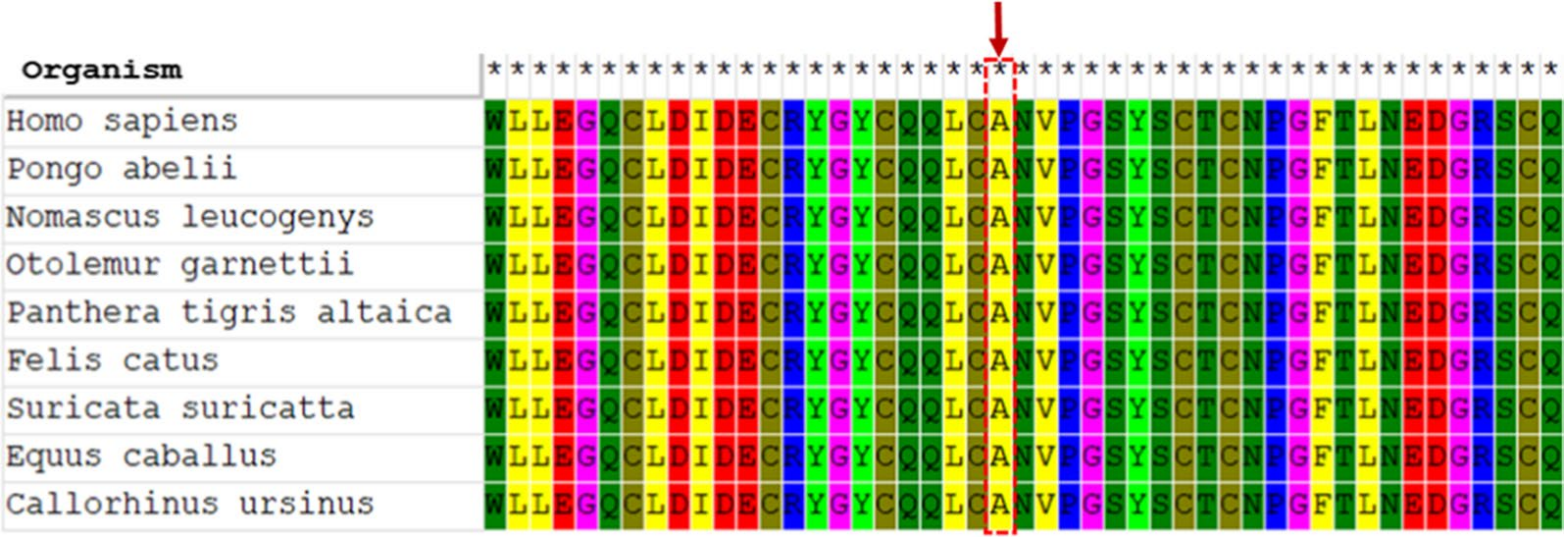
Amino acid alignment of A182P variation in different species showing a conservative amino acid

**Fig. 4:**
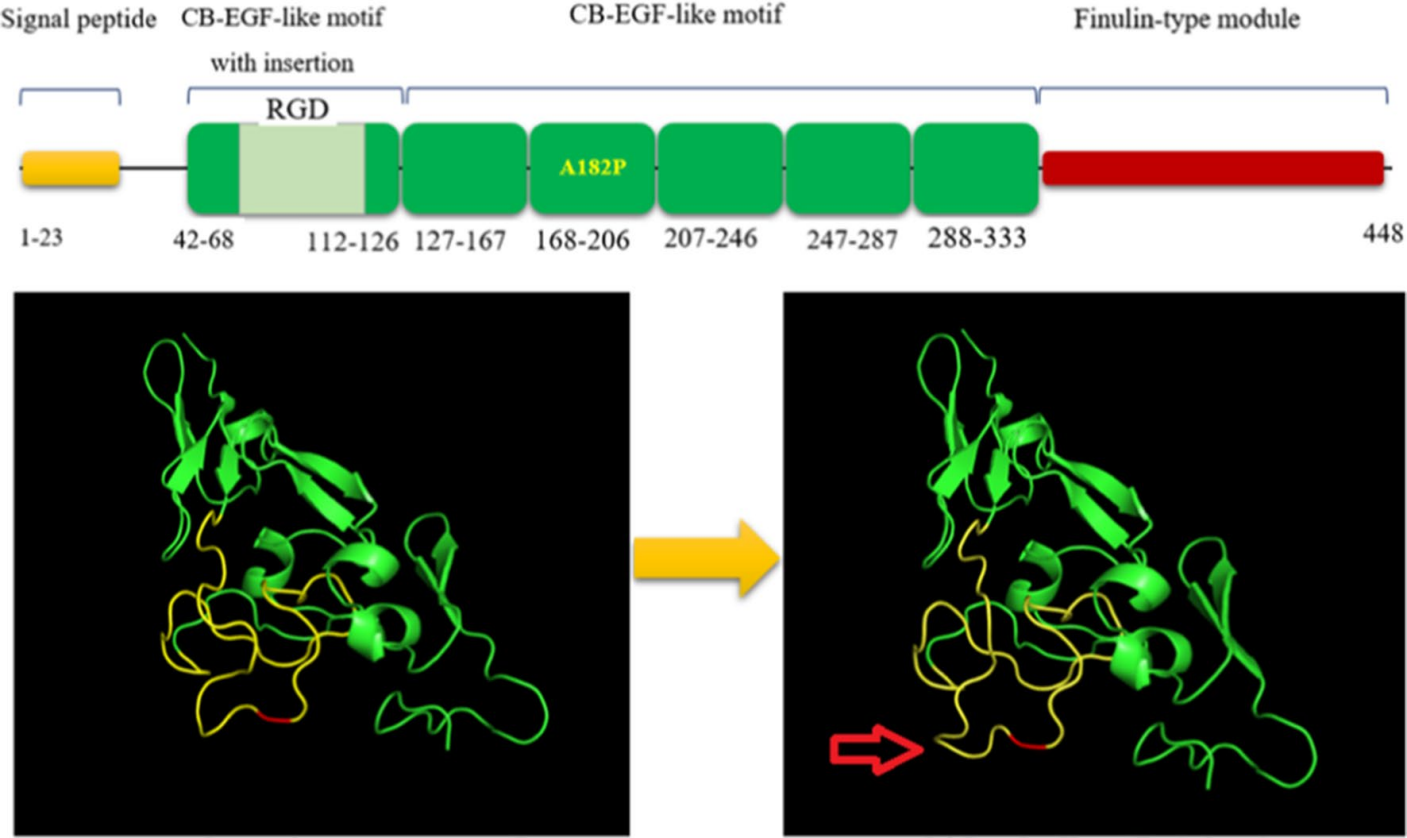
Schematic diagram of fibulin-5 structure. The protein domains (top) and 3D structure models for normal (left) and mutant (right) fibulin-5

Clinical diagnosis of affected individuals was on the basis of characteristic features, and they were suspected with different forms of CL at initial clinical evaluation. To diagnose a specific type of CL and identify inheritance pattern of the disease, the proband was analyzed through WES. A novel homozygous missense variant in exon 6 of *FBLN5* gene (c.G544C, p.A182P, reference sequence: NM_006329.3) was detected, suggesting the diagnosis of *FBLN5*-associated CL form. The variant is classified as "likely pathogenic" according to the ACMG guidelines (PM1, PM2, PP1, PP3) [15]. The nucleotide 544 in exon 6 and its corresponding amino acid Alanine is evolutionarily conserved across species from *Homo sapience* to *Callorhinus ursinus* (Fig. 3). This variant has not been reported in previous studies and gene variant public databases such as gnomAD, ClinVar, dbSNP, NCBI, EXOME variant databases, 1000 genome and HGMD. Based on most predictors including Mutation Taster, Ensembl variant effect predictor, and HANSA, this variant is pathogenic with SIFT and PolyPhen scores of 0.01 and 0.99, respectively. Protein structure predictors have shown that with substitution of Alanine with Proline in 182 position of FBLN5, the 3D structure of the protein has not changed significantly (Fig. 4).

**Fig. 5:**
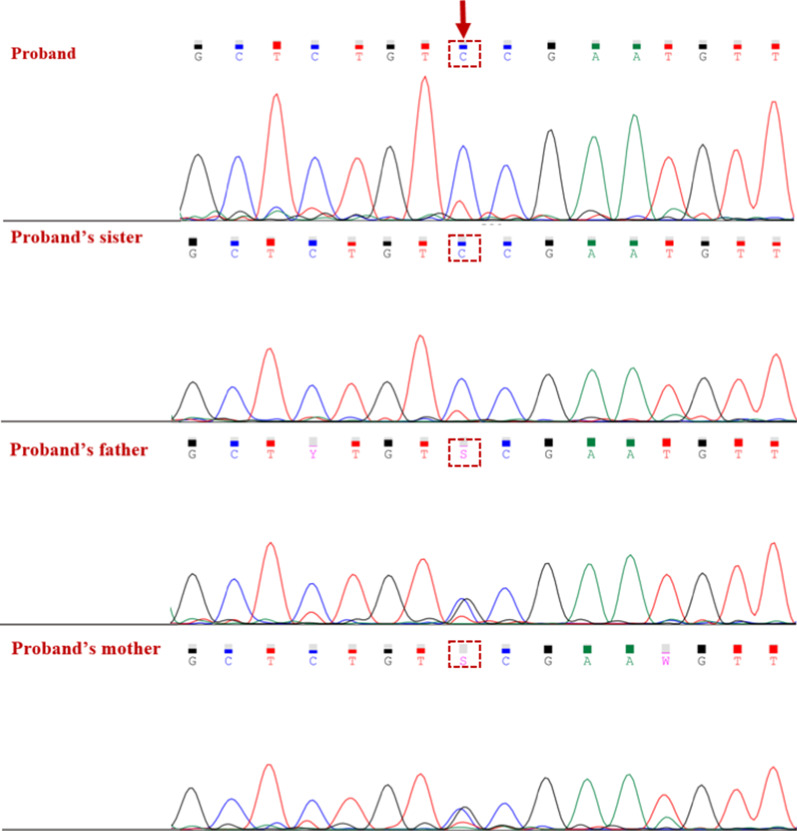
Co-segregation analysis. Variation analysis revealed that the proband and her sister were homozygous for c.G544C variation in FBLN5, whereas the parents were heterozygous carriers of the mutation

FBLN5-associated CL shows both autosomal dominant and recessive patterns of inheritance [16, 17]. Since none of consanguineous parents and also their relatives showed sign and symptoms of CL according to the pedigree, autosomal recessive pattern of inheritance was strongly suggested. To rule out any possibility of autosomal dominant inheritance especially in case of de novo mutation, co-segregation analysis of the variants was done. Our analysis showed that both parents and their mothers were heterozygous for this variation, stating the autosomal recessive mode of inheritance (Fig. 5).

## Discussion

The inherited form of CL can be caused by variations in diverse genes, which disrupt elastogenesis. In this study we assessed the clinical signs of monozygotic twin sisters and identified a novel homozygous missense variant in exon 6 of *FBLN5* gene through molecular analysis. Although the phenotype of *FBLN5*-related CL is broad, our patients did not show any pulmonary complications, gastrointestinal and genitourinary abnormalities. An interesting point about our patients is that only one of them showed VSD, suggesting that this type of CL has intrafamilial variability. Fibulin-5 is one of the integrin-binding members of the class II fibulin subfamily that is mostly found in the elastic-fibre-rich tissues such as skin, aorta, lung, and uterus [18, 19]. This glycoprotein is 66-kDa in size, contains 448 amino acids, including a signal sequence of 23 amino acids at the N-terminal, six calcium-binding EGF (cbEGF)-like motifs, and a C-terminal globular domain of 134 residues. An unusual long linker sequence with about 28 amino acids is present between the 4th and 5th cysteine residues of the first cbEGF motif. This domain also encompasses an RGD (arginine-glycine-asparatic acid) sequence that is evolutionally conserved [17, 20, 21].

The RGD motif is found in several matricellular and extracellular matrix (ECM) proteins such asosteopontin, fibronectin, thrombospondins, and vitronectin, and participates in cellular functions by binding with a subset of cell surface heteromeric integrins [20, 22]. The RGD sequence is the binding motif of fibulin-5 to human umbilical vein endothelial cells (HUVECs) [23]. This motif and the flanking domains in the N-terminal half of fibulin-5 act as mediators of cell attachment through interactions with αvβ3, αvβ5 and α9β1 integrin. Furthermore, the N-terminal half of fibulin-5 mediates attachment and spreading of primary aortic smooth muscle cells (SMCs) via binding to α5β1 and α4β1 fibronectin receptors but not to αvβ3 [24, 25]. However, after unmasking the RGD motif by reduction and alkylation in a direct protein interaction study, it has been shown that fibulin-5 could bind to αvβ3 [26]. Also, truncated protein with only the first cbEGF domain was not able to bind and spread SMCs, suggesting that other domains of fibulin-5 in the middle and C-terminus may be involved in this process. It is interesting that fibulin-5 after binding to α5β1 and α4β1 integrins is not able to activate downstream signaling. This protein has been proposed to be an inhibitor for fibronectin receptor-mediated signaling in a dominant-negative manner because of its dose-dependent antagonized role for fibronectin-induced stress fiber formation and focal adhesions in SMCs [20]. Considering the significance of the RGD domain in the assembly of elastic fibers, generating a D56E variation which known as a disrupting factor of the ECM to RGD-dependent integrins binding, showed completely normal elastic fibers assembly, suggesting that it is not necessary for formation of elastic fibers cell-surface binding of fibulin-5 [20, 26].

The cbEGF domains are found in most trans-membrane and ECM proteins and facilitate protein–protein interactions [27]. Through these domains, fibulin-5 binds to multiple ECM proteins including tropoelastin, latent TGF-β binding protein (LTBP)-2, lysyl oxidase like 1 (Loxl-1), Loxl-2, and 4, which are critical for elastic fiber assembly [28, 29]. For elastogenesis, a series of highly regulated steps including secretion and aggregation of the tropoelastin which is called coactivation, appropriate assembly and cross-linking of the tropoelastin, and then insoluble elastin organization into functional fibers are essential [30]. Fibulin-5 via binding to tropoelastin accelerates coacervation and also limits the maturation of elastin fragments which were coacervated [31, 32]. Another study has shown that in the skin of Fibulin5-null mice, the typical size of elastin aggregates was increased in comparison with wild-type mice [33]. According to these data, in the formation and maturation steps of coacervation process, fibulin-5 plays an important role in efficient control of coacervation and regulation of elastin aggregation optimal size for achieving accurate assembly and cross-linking of tropoealstin [20].

The identified novel homozygous missense variant in the current study leads to substitution of alanine 182 to proline in the third cbEGF. As mentioned above, the cbEGF motifs have critical role in elastic fiber assembly and variation of these domains can result in elastic fiber defects [34, 35]. Up to now, three different mutations namely I169T, R173H and G202R have been reported in the third cbEGF domain. The I169T variation decreases the secretion of the protein which compromises elastic fibre formation. The G202R that had been initially reported as a CL-related mutation was also detected in control groups in other study suggesting that this variant might not be pathogenic [35, 36]. The R173H variation has been detected in a Turkish family with affected child but the pathogenicity of the variant is obscure [16]. Other mutations in the adjacent domains such as V126M, C217R and S227P have been designated as pathogenic. The V126M not only causes hyperelasticity of the skin but also is associated with other diseases such as age-related macular degeneration and Charcot–Marie–Tooth disease type 1 [37]. Solid-phase binding and immunostaining studies in RFL-6 cells and patient-derived skin fibroblasts have shown that C217R and S227P are associated with reduction of fibulin-5-tropoelastin interaction. The second mutation was detected in two ethnically different families and results in a severe form of CL with internal organ involvement [38]. Considering the importance of the cbEGF motifs, it is obvious that pathogenic mutations in these motifs interfere with the fibulin-5 secretion and its matrix deposition which subsequently leads to diminished elastin polymerization. Considering that all the available variants predictors showed pathogenicity of A182P, we propose that this mutation may cause CL through disrupting of fibulin-5–tropoelastin interaction. Based on our 3D structure models both for wild and mutant fibulin-5 it seems that the A182P can contribute to the CL phenotype via the combined effect of lack of protein function and partly misfolding-associated toxicity. However, in a broader context, functional study of this variation is essential to uncover its pathogenicity and function of the cbEGF-3 domain.

Approximately all reported mutations of *FBLN5*, similar to the detected variation in this study follow autosomal recessive pattern of inheritance, and only one alteration, a tandem duplication of cbEGF 2–5 motifs, has showed autosomal dominant inheritance in a patient with mild form of CL [17]. Since point mutations such as S227P result in endoplasmic reticulum stress related to the recruitment of folding chaperones and increase patient-derived cells apoptosis, it appears that the recessive CL mechanisms are not only associated with a loss of fibulin-5 functional but also involve decreased cell survival [38]. Considering this, the large mutant protein can act in a dominant negative fashion and in case of homozygosity might result in severe form of CL.

## Conclusion

To sum up, we described clinical features of FBLN5-related CL and identified a novel variation in the cbEGF-3 domain. We also underlined the probable role and function of cbEGF motifs and proposed some possible mechanisms for recessive form of FBLN5-related CL. However, further functional studies are needed to confirm the pathogenicity of the variation and additional clinical and molecular investigations are indispensable to provide firm genotype–phenotype correlation and identify exact mechanisms which are involved in different types of this disorder.

## Data Availability

The datasets used and/or analysed during the current study are available from the corresponding author on reasonable request.

## Abbreviations

CL: Cutis laxa
AR: Autosomal recessive
VSD: Ventricular septal defect
ADCL: Autosomal dominant
ARCL: Autosomal recessive
XRCL: X-linked recessive
ALDH18A1: Aldehyde Dehydrogenase 18 Family Member A1
ELN: Elastin
FBLN5: Fibulin-5
ATP7A: ATPase copper transporting alpha
IVF: Vitro fertilization
WES: Whole exome sequencing
SMCs: Aortic smooth muscle cells
LTBP: Latent TGF-β binding protein
